# Supplementary material for: The interactome of intact mitochondria by cross-linking mass spectrometry provides evidence for coexisting respiratory supercomplexes
Source: Mol Cell Proteomics. 2017 Dec 8;17(2):216–32. doi: 10.1074/mcp.RA117.000470 (PMC5795388; doi:10.1074/mcp.RA117.000470)
Supplement: Supplemental Data [file supp_RA117.000470_133922_0_supp_23978_fzffwf.zip › spectra_annotation/mito_spectra_annotation/486-1-10-1-10-1.pdf]

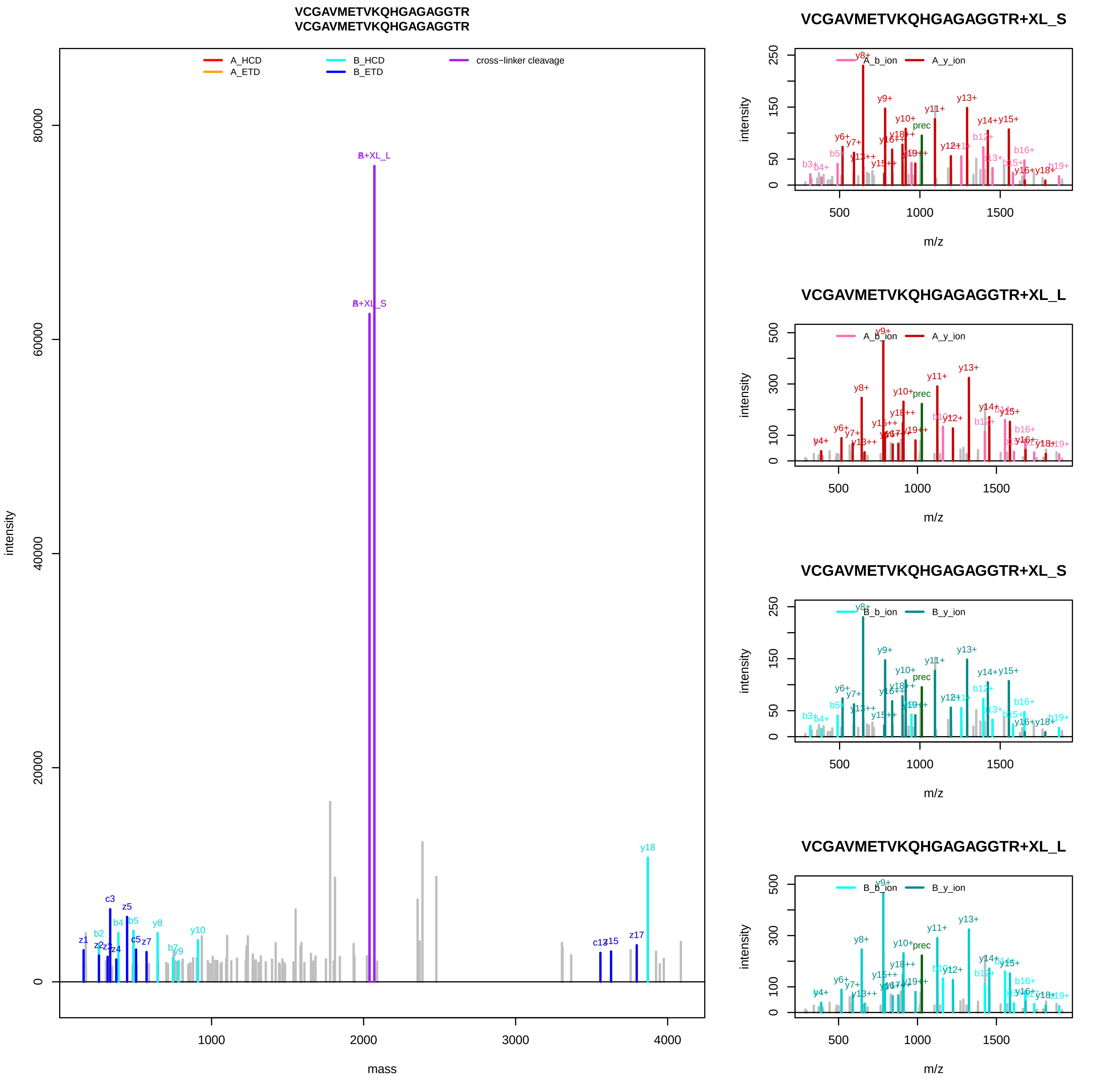

VCGAVMETVKQHGAGAGGTR
VCGAVMETVKQHGAGAGGTR
A_HCD
A_ETD
B_HCD
B_ETD
cross−linker cleavage
B+XL_L
B+XL_S
c3
z5
b5
b4
b2
z1
z4
c5
z7
y8
b7
y9
y10
c13
z15
z17
y18
intensity
mass
0
20000
40000
60000
80000
1000
2000
3000
4000
VCGAVMETVKQHGAGAGGTR+XL_S
A_b_ion
A_y_ion
VCGAVMETVKQHGAGAGGTR+XL_L
VCGAVMETVKQHGAGAGGTR+XL_S
B_b_ion
B_y_ion
VCGAVMETVKQHGAGAGGTR+XL_L
prec
m/z
intensity
0
50
150
250
100
300
500
500
1000
1500
